# Performance of Large Language Models in Lung Cancer Clinical Decision-Making: A Comparative Analysis Based on DeepSeek, Grok, and GPT

**DOI:** 10.7759/cureus.99026

**Published:** 2025-12-12

**Authors:** Yuyang Zhang, Dandan Yang, Yifan Shi, Ying Liu

**Affiliations:** 1 Department of Surgery, Jinzhou Medical University, Jinzhou, CHN; 2 Department of Internal Medicine, Jinzhou Medical University, Jinzhou, CHN; 3 Department of Ophthalmology, Jinzhou Medical University, Jinzhou, CHN; 4 Department of Research, Jinzhou Medical University, Jinzhou, CHN

**Keywords:** chatgpt, clinical decision-making, deepseek r1, grok 3, lung cancer

## Abstract

Large language models (LLMs) have reached a breakthrough in many aspects of imaging analysis and guideline mining, but not enough research has been conducted on applying them specifically to lung cancer applications. Three models were chosen, and corresponding questions that highlighted specificity towards the diagnosis of lung cancer were proposed with the goal of providing data to increase confidence and improve recommendations for transforming AI-driven clinical care for lung cancer. In this study, five clinical domains were defined. Each question was individually uploaded to the models, and responses were evaluated by three thoracic surgery experts based on accuracy, completeness, and practicality. DeepSeek-R1, Grok-3, and GPT-4.5 showed different levels of results when it came to providing clinical support for lung cancer. Regarding their responses to clinical questions, Grok-3 had a much longer average response and better performance scores. Subgroup analyses further showed that Grok-3 scored the highest of all five domains. Additionally, the confidence scores on recognizing images in the text of Grok-3 were the highest; most mistakes occurred in the differential diagnosis of special cases, whereas DeepSeek and GPT all gave preference to rarer or infectious diseases.

## Introduction

Lung cancer remains the biggest cancer killer globally, with the disease burden continuing to rise. According to GLOBOCAN 2022 estimates, there were approximately 2.4 million new cases of lung cancer and 1.8 million deaths globally [1]. In China, there were approximately 733,300 cancer deaths associated with lung cancer in 2022, accounting for 28.5% of all cancer deaths due to malignancies in that year [2]. The clinical management of lung cancer is complicated by multiple factors, including chronic smoking and carcinogenic air pollution exposure [3-5], early-stage manifestation, complex pathologic stages, and a large number of different kinds of personalized treatment methods, making diagnosis and treatment decision-making extremely difficult. With the help of the rapidly developing technology and strong interest of the medical field in AI, much research [6-8] has shown tremendous potential of LLMs that could offer good assistance for medical decision support [9-11], structured medical records [12,13], assisted diagnosis and treatment [14], disease prevention [15], and personalized care through sophisticated natural language processing capability. Previous studies focusing on general medical knowledge evaluation outweigh those specific medical field knowledge assessments of lung cancer [16,17]. Empirical studies evaluating clinical decision support (CDS) systems for specific clinical problems such as lung cancer are limited, highlighting the need for systematic evaluation [18]. The primary aim of this study was to systematically evaluate the accuracy, completeness, and clinical practicality of three advanced large language models, DeepSeek R1, Grok-3, and GPT-4.5, in simulated lung cancer clinical decision-making scenarios. Secondary aims included assessing performance variations across five key clinical domains and evaluating the models’ capability to interpret standardized radiological text descriptions.

## Materials and methods

Clinical trial number

A clinical trial number does not apply in this case.

Ethics

Approval from the ethics committee was not required since no patients were involved in our study.

Study design

The 21 simulated cases were constructed by a panel of three thoracic surgeons and two radiologists, based on their clinical expertise and actual patients. Cases were designed to represent a spectrum of histologies, stages, and treatment phases: eight squamous cell carcinomas, four small cell lung cancers, and nine adenocarcinomas; TNM staging: five Stage I, five Stage II, six Stage III, and five Stage IV cases. The treatment phases were distributed as follows: five initial diagnoses, six chemotherapy, six postoperative, and four recurrences. The simulated study consisted of 11 males and 10 females. Standardized textual descriptions of CT findings were formulated for each case by two radiologists, using a structured template (e.g., location, size, morphology, lymph nodes) based on lung-RADS descriptors. Any discrepancies were resolved by consensus. A total of 21 standardized lung cancer-related clinical questions were posed to the three LLMs: DeepSeek R1, Grok-3, and ChatGPT-4.5. Five inquiry directions were established: 1) diagnosis and staging, 2) treatment decisions, 3) complication management, 4) prognosis assessment, and 5) patient education and psychological support. Each case was randomly assigned one inquiry direction. LLM interaction protocol: all questions were posed via the official web interfaces (DeepSeek R1, Grok-3, ChatGPT-4.5) from April 2, 2025, to April 10, 2025. A standardized system prompt was used: "As a thoracic surgery expert/radiologist, based on the following clinical/imaging findings, what is the most likely diagnosis?" followed by the case. Model parameters were left at their default settings. Each question was initiated in a new session to prevent context carryover. The responses were converted to plain text and stripped of the specific LLM features. DeepSeek R1, Grok-3, and ChatGPT-4.5 were designated AI I, AI II, and AI III, respectively, yielding 63 responses in total, all converted to plain text format. The three thoracic surgery experts had a mean of eight years of post-fellowship experience. They were blinded to the identity of the LLM generating each response (Fleiss‘ kappa: 0.36). Table 1 presents the answer evaluation form, featuring each response on an individual sheet, arranged in random order, and submitted to three thoracic surgery experts for assessment based on accuracy, completeness, and practicality. Parallel to the above, diagnostic evaluations with standard textual descriptions of CT images were conducted for each question. The process was repeated three times, and each diagnostic evaluation dialogue was reopened after one complete question, facilitating their repeated evaluations on detecting the CT images. 

**Table 1:** Evaluation of medical answers

Accuracy	
Level 5	The answer is fully consistent with guidelines and expert consensus, containing no erroneous information
Level 4	The answer aligns with guidelines and expert consensus, but the conclusion is incomplete
Level 3	The main conclusion of the answer is correct, but there are minor errors
Level 2	Some parts of the answer are correct, but there are serious errors
Level 1	The answer is completely incorrect or contradicts the guidelines
Completeness	
Level 5	The answer comprehensively addresses the medical query and provides additional medical information and insights
Level 4	The answer accurately addresses the medical query but does not provide additional medical information and insights
Level 3	The answer provides basic responses to the medical query but lacks depth and detail
Level 2	The answer only partially addresses the medical query or misses key points
Level 1	The answer has serious deficiencies in addressing the key points of the medical query or is irrelevant
Practicality	
Level 5	The answer can be directly applied to clinical decision-making without any modification
Level 4	The answer requires minor modifications to be applied to clinical decision-making
Level 3	The answer requires major modifications to be applied to clinical decision-making
Level 2	The answer is too vague to be applied to clinical decision-making
Level 1	The answer is completely incorrect and cannot be applied to clinical decision-making

Statistical analysis

Data analysis was conducted using IBM Corp. Released 2021. IBM SPSS Statistics for Windows, Version 27. Armonk, NY: IBM Corp., while means±standard deviation (SD) values represented expert judgments of each response; graphs used GraphPad Prism (10.4.2) and OriginPro (2024). One-way ANOVA, Tukey’s post-hoc test, and Dunn’s post-hoc test were used to perform the response duration tests to compare the accuracy, completeness, and practicability of the three LLMs. Adjusted p-values were corrected using the Bonferroni correction procedure, and a p-value <0.05 was taken as statistically significant.

## Results

Baseline characteristics of the lung cancer cohort

Table 2 summarizes the clinical characteristics of the lung cancer cohort. The pathological types were squamous cell carcinoma (n=8, 38.1%), small-cell lung cancer (n=4, 19%), and adenocarcinoma (n=9, 42.9%). TNM staging distribution: stage I (n=5, 23.8%), stage II (n=5, 23.8%), stage III (n=6, 28.6%), and stage IV (n=5, 23.8%). Treatment phases: initial diagnosis (n=5, 23.8%), chemotherapy (n=6, 28.6%), postoperative (n=6, 28.6%), and recurrence (n=4, 19%). Sex distribution: male (n=11, 52.4%) and female (n=10, 47.6%).

**Table 2:** Patient characteristics of the simulated cohort

Feature	Category		Number of Cases (n=21)	Percentage (%)	
Pathological Type	Squamous Cell Carcinoma		8	38.1	
	Small Cell Lung Cancer		4	19.0	
	Adenocarcinoma	EGFR mutation	3	14.3	42.9
		ALK fusion	2	9.5	
		KRAS mutation	2	9.5	
		no mutation	2	9.5	
TNM Staging	Stage I		5	23.8	
	Stage II		5	23.8	
	Stage III		6	28.6	
	Stage IV		5	23.8	
Treatment Phase	Initial Diagnosis		5	23.8	
	Chemotherapy		6	28.6	
	Postoperative		6	28.6	
	Recurrence		4	19.0	
Sex distribution	Male		11	52.4	
	Female		10	47.6	

Response length comparison across LLMs

Table 3 shows LLM responses to lung cancer questions. The mean word count ± SD is 495.86 ± 63.75 for DeepSeek R1, 706.81 ± 98.47 for Grok-3, and 441.19 ± 71.56 for GPT-4.5. With characters being counted, DeepSeek R1 had an average count of 3529.57 ± 445.58, Grok-3 was 4848.48 ± 555.64, and GPT-4.5 was 2956.52 ± 424.84.

**Table 3:** Response lengths to lung cancer-related questions

	Response Length (Words)			Response Length (Characters)		
LLM	Mean ± SD	Max	Min	Mean ± SD	Max	Min
DeepSeek R1	495.86 ± 63.75	627	357	3529.57 ± 445.58	4501	2344
Grok-3	706.81 ± 98.47	939	555	4848.48 ± 555.64	6480	3606
GPT-4.5	441.19 ± 71.56	587	311	2956.52 ± 424.84	3762	2103

Overall performance of LLMs in clinical questions

**Figure 1:**
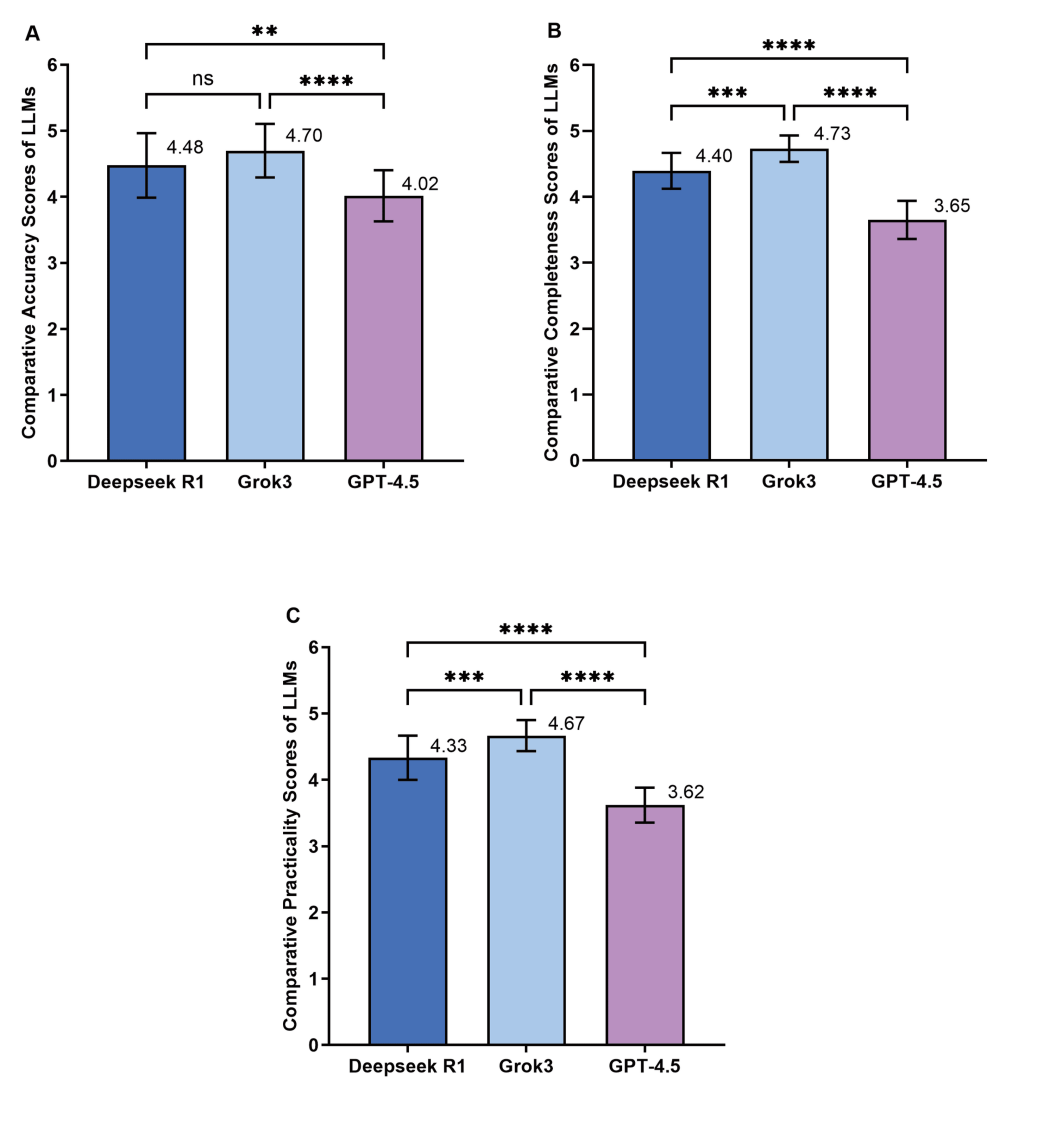
Comparative assessment of accuracy, completeness, and practicality in responses to lung cancer-related queries across three large language models Accuracy, completeness, and practicality scores of responses from DeepSeek R1, Grok-3, and ChatGPT-4.5 across 21 cases, as evaluated by three thoracic surgery experts: (A) mean overall accuracy scores, (B) mean overall completeness scores, and (C) mean overall practicality scores of their responses across the 21 cases. In the statistical analysis, Dunn’s post-hoc test was used. A p-value of less than 0.05 was considered statistically significant. Number, *p＜0.05, **p＜0.01, ***p＜0.001, ****p ＜0.0001

Figure 1 shows the responses of the three language models to the lung cancer questions. The more specific quantification for the LLM accuracy shown in the chart, Grok-3 performed best, at 4.70 ± 0.41 compared to GPT-4.5 (4.02 ± 0.39; Dunn’s post hoc test p <0.0001). Complete accuracy was achieved by Grok-3, with a score of 4.73 ± 0.20, outperforming DeepSeek R1 (4.40 ± 0.27; p = 0.0002) and GPT-4.5 (3.65 ± 0.29; p < 0.0001). In practice, Grok-3 scores 4.67 ± 0.24 points, followed by DeepSeek R1 at 4.33 ± 0.33 points (p = 0.0006) and GPT-4.5 at 3.62 ± 0.26 points (p < 0.0001).

Domain performance of LLMs in clinical subgroups

**Figure 2:**
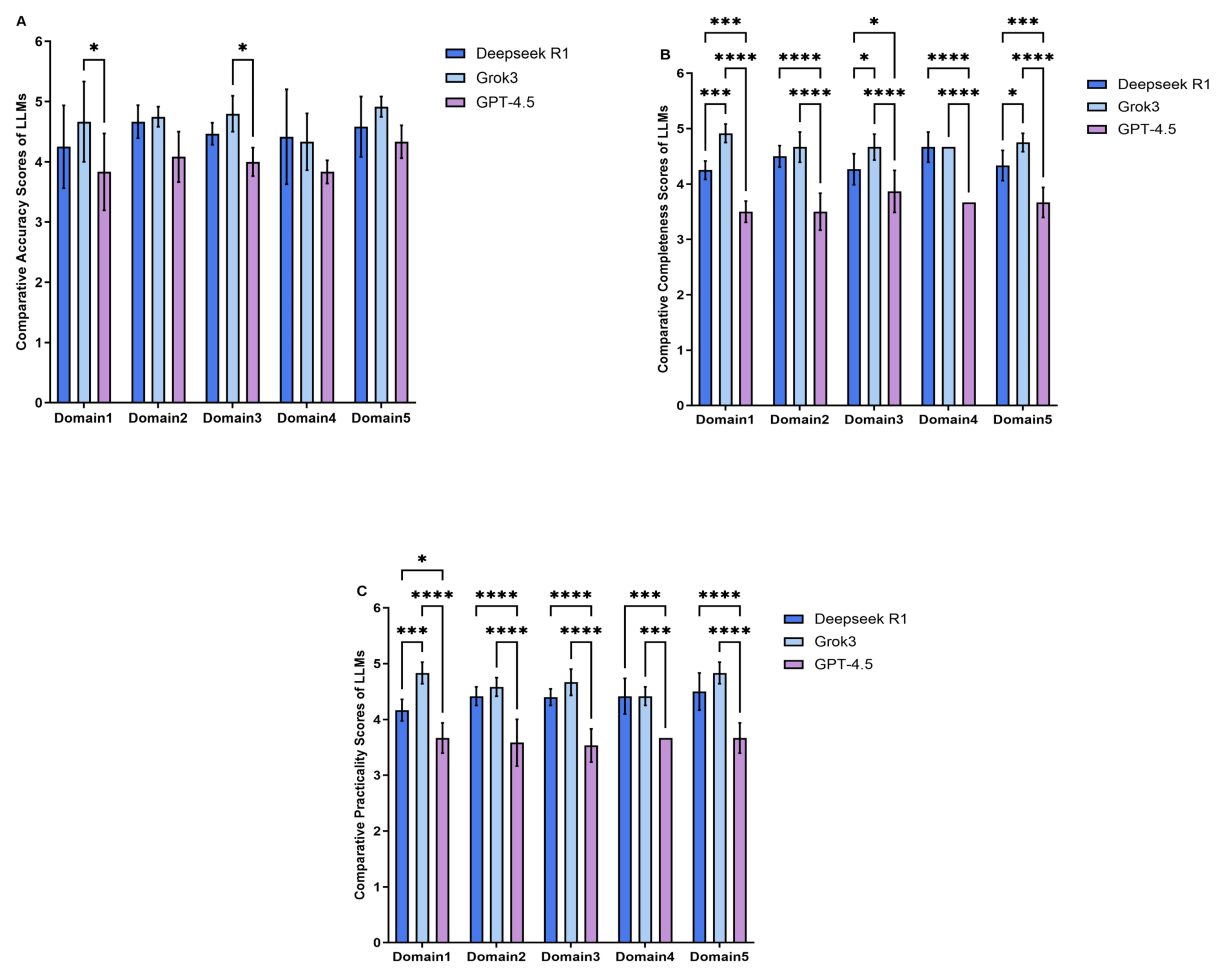
Performance across five clinical domains in lung cancer-related query responses by three large language models Domain 1: Diagnosis and staging; Domain 2: Treatment decisions; Domain 3: Complication management; Domain 4: Prognosis assessment; Domain 5: Patient education and psychological support. (A) Accuracy scores of DeepSeek R1, Grok-3, and ChatGPT-4.5 in the five clinical areas. Panel (B) Completeness scores of DeepSeek R1, Grok-3, and ChatGPT-4.5 in the five clinical areas. (C) Practicality scores of DeepSeek R1, Grok-3, and ChatGPT-4.5 in the five clinical areas. In the statistical analysis, Tukey’s post-hoc test and Dunn’s post-hoc test were used. A p-value of less than 0.05 was considered statistically significant. Number, p＜0.05, *p＜0.01, ***p＜0.001, ****p＜0.0001.

Figure 2 shows the subgroup analyses of the results according to the question categories in which Grok-3 had the best overall accuracy over all five domains, surpassing both DeepSeek R1 and GPT-4.5, which showed slightly higher accuracy for prognostic predictions compared with DeepSeek R1 (p >0.05). However, in completeness, Grok-3’s maximum performances were recorded for diagnosis and staging, treatment decisions, complication management, and patient education and psychological support, comparable to that of DeepSeek R1 for the same purpose of providing prognostic predictions. The accuracy recorded by Grok-3 was notably higher than that recorded by DeepSeek R1 (p = 0.0009) and GPT-4.5 (p < 0.0001) on the basis of diagnosing and staging questions; the same tendency was true for practicality questions (p < 0.01).

Radiological text recognition capabilities

LLMs performed well when analyzing the text within the CT images. DeepSeek R1 and Grok-3 exhibited higher consistency (90.5%; 19/21) than GPT-4.5 (81.0%, 17/21). Among all participants, Grok-3 was the most accurate (93.7%, 59/63), with some errors, such as some unusual cases being misclassified. For instance, patient 2 was misdiagnosed with tuberculosis, fungal infection, or sarcoidosis; patient 17 was given two correct classifications, but tuberculosis was mistakenly identified. On the contrary, DeepSeek R1 had higher accuracy rates (77.8%; 49/63), and more errors occurred (22.2%; 14/63) for occasional rare disease misclassifications, such as patient 8 being mistakenly classified as pulmonary Langerhans’ cell histiocytosis (PLCH) and patients 3, 16, and 19 having two errors. Patient 16 and patient 21 were diagnosed with two errors of lymphangioleiomyomatosis (LAM) and one error of chronic obstructive pulmonary disease (COPD). GPT-4.5 also reached an accuracy of 85.7% (54/63), but its error rate was slightly higher, being around 14.3% (9/63) for these five patients, including tuberculosis, sarcoidosis, and pulmonary fibrosis.

## Discussion

This study assessed the language quality, clinical efficacy, and multimodal information processing capabilities of three leading LLMs in the context of lung cancer clinical scenarios. This evaluation was conducted through case-based inquiries and standardized CT image text descriptions, in conjunction with expert assessments, thereby highlighting their potential medical applications and areas for improvement. All the models generated extensive positive responses. Reasoning-capable models exhibited high accuracy, completeness, and deep reasoning comparable to clinical experts in generating medical information and performing logical reasoning for actual lung cancer cases. This suggests their potential as clinical decision support tools. These findings are consistent with previous research indicating the utility of LLMs in medical education, where they match human expert performance in USMLE tests and approaches or achieve expert levels in diagnosis, decision support, education, and patient communication. However, pattern-recognition-based models did not meet this standard, lacking comparable accuracy or comprehensiveness. Subgroup analysis showed that Grok-3 consistently led across all five domains, with DeepSeek closely following in most areas, indicating scenario-specific strengths and supporting the development of modular medical LLM systems. Grok-3’s superior performance may stem from its chain-of-thought reasoning approach, which systematically evaluates possibilities, unlike pattern-matching-reliant open-source models that are unsuitable for autonomous clinical decisions [19-21].

In terms of CT image text analysis, the consistency rates between DeepSeek R1, Grok-3, and the radiologist were significantly higher than GPT-4.5; therefore, they had a certain application value in the pre-exam triage and could relieve radiologists’ workload and improve the efficiency of examinations. However, there was a special kind of diagnosing error in which DeepSeek R1 made errors in atypical cases, Grok3 made mistakes when facing rare diseases, and GPT-4.5 made errors in non-specific imaging features, and they are not typical “AI hallucinations” but express that they could not understand medical knowledge comprehensively due to the so-called lack of overall information connected to specific contexts of concepts [22,23]. The result highlighted that there needed to be professional attendants and particular attention for both errors and accuracy; it needs more caution. Even reinforcement learning could not make GPT-4.5 work perfectly for complicated tasks, including staging or treatment for rare diseases, leading to possible errors or incompleteness of outputs. Relevant LLMs and other critical points related to the clinical applications of LLMs in lung cancer deserve further exploration. While these models have potential as decision support systems [24], they cannot replace clinicians, especially when dealing with special cases outside the standard guidelines, and without continuous updates, LLMs would always be lagging behind clinical progress. Therefore, there is a risk of the existence of improper guidance when an untrained model is used. These models can be optimized in this way by connecting to the diagnostic workflow, training them on images and laboratory exams, and improving the conversational style.

This research has several limitations: a limited amount of data makes it impossible for this study to have great generalizability. The test set consists mainly of the classic cases of lung cancer, which underestimates its performance. Furthermore, although CT descriptions are based on genuine and realistic cases, their downstream clinical impact (e.g., user experience and diagnostic efficiency) has not been explored. Future work should explore training domain-specific data sets to improve the LLM performance and testing features in a prospective evaluation.

We suggest establishing benchmarks for different lung cancer types, complex cases, etc., which involve more types of multimodal data such as imaging, pathology, molecular testing, and so on, to build a standard set, not only for common cases but also for uncommon cases, in order to make models learn how to deal with complex scenarios [25-27]. If we can provide better prompts and ask better questions about lung cancer diagnosis to our model (using prompt engineering), the outcomes will obviously be more optimized [28]. Future development of LLMs is to focus on providing auxiliary rather than decision-making services to doctors. They can do what humans think machines can do better, so that machines and humans can help each other to improve diagnosis and treatment quality.

## Conclusions

LLMs show varied performance on lung cancer support. Grok-3 demonstrated statistically superior performance across multiple metrics. This suggests its potential for further investigation as a clinical support tool. Meanwhile, LLMs show consistent results for typical lesions in CT texts but poor results in special situations, causing misclassification as atypical or rare diseases, emphasizing that improved pathological skills and more extensive data knowledge are necessary for easy prediction. Although LLMs can optimize oncology workflows, an LLM-based clinical framework that combines the expertise of specialists and expert clinical decision-making with multimodal data is still required, especially for ambiguous cases, before there is a sufficiently strong enough tool that performs the required analysis of complex characteristics.

## Figures and Tables

**Figure 3:**
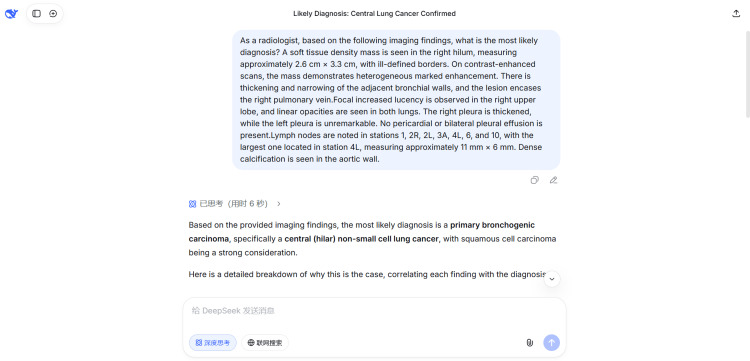
DeepSeek R1 answers lung cancer imaging questions

**Figure 4:**
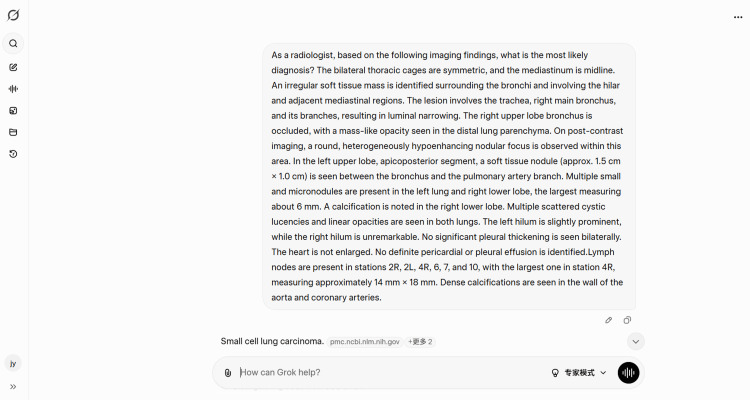
Grok-3 answers lung cancer imaging questions

**Figure 5:**
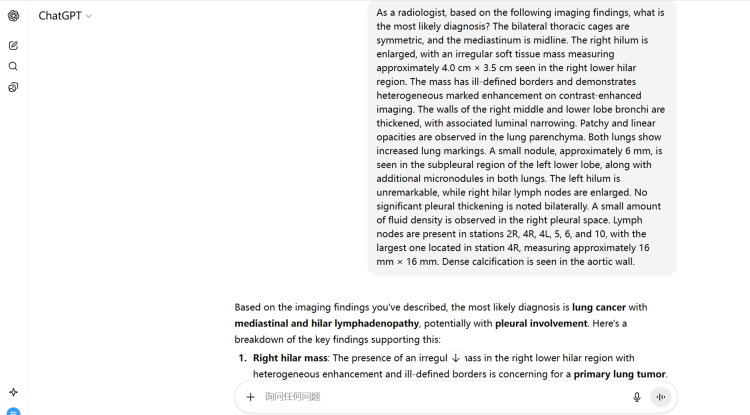
GPT-4.5 answers lung cancer imaging questions
