# Correction: Performance of Large Language Models in Lung Cancer Clinical Decision-Making: A Comparative Analysis Based on DeepSeek, Grok, and GPT

**DOI:** 10.7759/cureus.c386

**Published:** 2025-12-22

**Authors:** Yuyang Zhang, Dandan Yang, Yifan Shi, Ying Liu

**Affiliations:** 1 Department of Surgery, Jinzhou Medical University, Jinzhou, CHN; 2 Department of Internal Medicine, Jinzhou Medical University, Jinzhou, CHN; 3 Department of Ophthalmology, Jinzhou Medical University, Jinzhou, CHN; 4 Department of Research, Jinzhou Medical University, Jinzhou, CHN

The ethics statement of this article has been corrected to include the following: "This work was supported by the Undergraduate Innovation and Entrepreneurship Training Program of Jinzhou Medical University (Grant No. X202410160119)."

